# “Neuromyths” and Multiple Intelligences (MI) Theory: A Comment on Gardner, 2020

**DOI:** 10.3389/fpsyg.2021.720706

**Published:** 2021-08-06

**Authors:** Luc Rousseau

**Affiliations:** Department of Psychology, Laurentian University, Greater Sudbury, ON, Canada

**Keywords:** education, neuromyths, intelligence, multiple intelligences theory, matching hypothesis

## Introduction

Neuromyths are misconceptions about the brain and learning. The most pervasive neuromyths contain a “kernel of truth” (Grospietsch and Mayer, 2018). For instance, consider the following popular neuromyth: *People are either “left-brained” or “right-brained,” which helps to explain individual differences in learning*. On the one hand, classical neuroscience findings did provide solid basic evidence that the human brain displays a certain degree of functional hemispheric lateralization (Gazzaniga et al., 1962, 1963). However, on the other hand, the idea of a “dominant” cerebral hemisphere is not supported by neuroscience (Nielsen et al., 2013). Due to fatal mutations from kernels of truth, neuromyths are typically defined as distortions, oversimplifications, or abusive extrapolations of well-established neuroscientific facts (OECD, 2002; Pasquinelli, 2012; Howard-Jones, 2014).

In the past decade, numerous surveys have been conducted in more than 20 countries around the world to measure the prevalence of neuromyth beliefs among educators (Torrijos-Muelas et al., 2021). A large-scale survey conducted in Quebec, Canada, by Blanchette Sarrasin et al. (2019) revealed that 68% of teachers *somewhat* or *strongly* agreed (rating of 4 or 5 on a 5-point scale) with the following neuromyth statement:

*Students have a predominant intelligence profile, for example logico-mathematical, musical, or interpersonal, which must be considered in teaching*.

This is not an idiosyncratic case in the field (see Table 1). In another survey conducted in Spain, Ferrero et al. (2020) reported that teachers gave an average rating of 4.47 [on a 5-point scale, from 1 (*definitely false)* to 5 *(definitely true*)] to a closely similar neuromyth statement:

*Adapting teaching methods to the “multiple intelligences” of students leads to better learning*.

**Table 1:** Prevalence of beliefs, among educators, about the false claim that tailoring instruction to pupils' MI intelligence profiles promotes learning, in different countries around the world.

**Survey**	**Country**	**Neuromyth statement**	**Sample**	**Finding**
Blanchette Sarrasin et al. (2019)	Quebec, Canada	*Students have a predominant intelligence profile, for example logico-mathematical, musical, or interpersonal, which must be considered in teaching*.	In-service teachers (*n* = 972)	68% of teachers *somewhat* or *strongly* agreed (rating of 4 or 5 on a 5-point scale) with the statement.
Craig et al. (2021)	Canada/USA	*Basing instructional strategies on multiple intelligences (e.g., linguistic, musical, and interpersonal intelligence) is not supported by research. [True]* (inverted item)	In-service teachers (*n* = 253)	24.9% of correct answers (*True*).
Ferrero et al. (2020)	Spain	*Adapting teaching methods to the “multiple intelligences” of students leads to better learning*.	In-service teachers (*n* = 45)	*M* = 4.47 on a 5-point scale, from 1 (*definitely false*) to 5 (*definitely true*).
Rogers and Cheung (2020)	Hong Kong	*Good teaching requires aligning instruction to the multiple intelligences ofstudents*.	Pre-service teachers (*n* = 65)	*M* = 4.57 on a 6-point scale, from 1 (*strongly disagree*) to 6 (*strongly agree*).
Ruhaak and Cook (2018)	USA	*Teaching to multiple intelligences*.	Special education pre-service teachers (*n* = 129)	90% of prospective teachers will *probably* or *definitively* implement the instructional practice (rating of 3 or 4 on a 4-point scale).

The opening survey statement from Blanchette Sarrasin et al. (2019) caught Howard Gardner's attention, because it clearly draws from his Multiple Intelligences (henceforth MI) theory (Gardner, 1983). In a recent paper, Gardner (2020) says he was disturbed by this so-called “neuromyth,” both because it says nothing about the brain, and because it is not an idea that he has put forth or defended. On that basis, Gardner (2020) argues that MI theory does not qualify as a neuromyth. According to the author of *Frames of Mind*, some years ago, there may have been merit in exposing neuromyths, but the practice has gone too far and has now become problematic rather than helpful.

In this opinion paper, I first challenge Gardner's (2020) view that MI theory contains no “neuro.” Then, I highlight the fact that Gardner and his research team spent an entire decade, through the *Spectrum Project*, contemplating the hypothesis—embedded into the opening survey statement—that matching modes of instruction to MI intelligence profiles promotes learning. When taken for granted, such an unproven research hypothesis is considered as a false belief—a neuromyth derived from MI theory. Then, I argue that research aimed at testing the MI–instruction “matching” hypothesis is still hampered by a lack of satisfactory measures of MI intelligence profiles. Finally, I expose how Gardner's (2020) position may, paradoxically, entertain the “problematic” neuromyth. To foster a more constructive dialog between scientists and educators, I follow Gardner's (2020) advice to properly qualify (i.e., to debunk) the survey statement, in terms of both robustness and caveats.

## Biological Basis for Specialized Intelligences

Gardner (2020) states that “there is no mention of the brain” in his original work, insisting that “MI is a psychological theory, pure and simple” (p. 3). Because MI theory contains no “neuro,” he claims, there is no reason why it should be associated with the “provocative and contentious neuromyth” term. However, Gardner has typically called MI “a psychobiological theory: psychological because it is a theory of the mind, biological because it privileges information about the brain, the nervous system, and ultimately, [he] believe[s], the human genome” (Gardner, 2011b, p. 7). In the opening chapters of *Frames of Mind*, after disposing of traditional, IQ theories of intelligence, Gardner (1983) draws from brain science of the day to posit the basic premise of MI theory—that intelligences are distinct computational capacities that have emerged, over the course of evolution and across cultures, from the human cerebral cortex:

We find, from recent work in neurology, increasingly persuasive evidence for functional units in the nervous systems. There are units subserving microscopic abilities in the individual columns of the sensory or frontal areas; and there are much larger units, visible to inspection, which serve more complex and molar human functions, like linguistic or spatial processing. These suggest a biological basis for specialized intelligences (p. 57).

Such neurological evidence led Gardner (1983) to include potential isolation by brain damage as one of eight criteria—actually “the single most instructive line of evidence” (p. 63)—to define an intelligence. Critical insights for MI theory also came from Gardner's earlier neuropsychological research conducted in the 1970s on brain-damaged patients suffering from aphasia (Gardner, 2011b, 2016). Consistent with intelligences as *biopsychological potentials to process information*, Davis et al. (2011) noted that it would be “desirable to secure an atlas of the neural correlates of each of the intelligences” (p. 495) and current neuroscientific investigations of MI theory are undergoing in that direction. For instance, a brain lesion restricted to the left parietal lobe would selectively impair the capacity to discriminate living from non-living entities, i.e., *naturalistic intelligence* (Shearer and Karanian, 2017).

But even with no “neuro” at all, MI theory would still qualify as a potential source of neuromyths, as *any* scientific theory could—be it psychological, neurological, or a mix of both. Myths may have nothing to do with the brain, but are, nonetheless, myths. Over time, the term “neuromyth” has become a common umbrella to a wide range of unsubstantiated claims, especially in the education field. Some of those claims clearly evoke the brain (e.g., *We only use 10% of our brain)*, while others do not (e.g., *Listening to Mozart's music makes children smarter*). Would it be more appropriate to drop the “neuro” prefix and collectively call them “edumyths”? Actually, it does not matter. They are myths.

Above all, the primary aim of MI theory was to expand the traditional, narrow IQ concept of intelligence to the whole spectrum of brain computational powers, *not* to provide brain-based educational recommendations. The basic idea of MI theory is that *Homo sapiens* is biologically endowed with a set of relatively autonomous mental tools (termed “intelligences”) that can be activated to solve problems or to fashion products that are of cultural value. MI theory posits that every individual has, at their disposal, a *full* intellectual profile of eight intelligences. From one individual to another, some intelligences exhibit low, some exhibit average, and some others exhibit strong biopsychological potentials, but the whole MI intelligence profile—a spectrum of brain computational powers working in synergy—is mobilized to adapt *Homo sapiens* to newly encountered, culture-bound situations.

## The Elusive Quest for Optimal Matching

Unlike Gardner's (2020) allegation, the claim in the opening survey statement is not that MI theory is a neuromyth. There has been considerable progress in brain science over the past four decades, and neurological underpinnings of the original rendition of MI theory (Gardner, 1983) might need an update (Gardner, 2016), but MI theory is still a plausible, legitimate scientific theory of intelligence. The false claim in the opening survey statement is that tailoring instruction to pupils' MI intelligence profiles promotes learning. Gardner (2020) states that he has “gone to great pains to emphasize that even if the theory is plausible, no educational recommendations follow directly from it” (p. 3). However, since the inception of MI theory some 40 years ago, regarding applications of MI theory in education, Gardner oscillates between two views: the “Rorschach” view and the “matching” view.

According to the “Rorschach” view, defended by Gardner (2020), no direct educational implications derive from research findings. Cultural values always interface the leap from science to practice. In this view, MI theory is a *catalyst* for reflection on a pluralistic, rather than a unitary, view of intelligence (Gardner, 1995a). To use Gardner's (2006) analogy, from the teachers' standpoint, MI theory is an educational Rorschach test, a backdrop “to support almost any pet educational idea that they had” (Gardner, 2011b, p. 5). MI theory implies only two non-prescriptive teaching practices: “individualizing” and “pluralizing.” By using multiple “entry points” (presenting the teaching materials in more than one way), teachers might activate all intelligences and foster optimal learning, “since some individuals learn better through stories, others through work of art, or hands-on activities” (Gardner, 2011b, p. 7).

According to the alternative, “matching” view, clearly embedded in the opening neuromyth statement, Gardner (2020) states that it is “not an idea that [he] has put forth or defended” (p. 2). However, in the closing chapter of *Frames of Mind*, from a purely speculative and prospective standpoint, Gardner (1983) is quite sympathetic to the idea of matching teaching materials and modes of instruction to MI intelligence profiles:

Educational scholars nonetheless cling to the vision of the optimal match between student and material. In my own view, this tenacity is legitimate: after all, the science of educational psychology is still young; and in the wake of superior conceptualizations and *finer measures* [emphasis mine], the practice of matching the individual learner's profile to the materials and modes of instruction may still be validated. Moreover, if one adopts M.I. theory, the options for such matches increase: as I have already noted, it is possible that the intelligences can function both as subject matters in themselves and as the preferred means for inculcating diverse subject matter (p. 390).

Albeit speculative, and much to Gardner's surprise, these few lines have attracted tremendous interest in the education field. But testing the matching hypothesis required, in the first place, “finer measures” of MI intelligence profiles. Gardner (1992) proposed, as an alternative to IQ-like paper-and-pencil (standardized) intelligence tests, natural observations of *Homo sapiens* freely evolving in ecologically valid, culturally meaningful contexts. For instance, to measure *spatial intelligence*, “one should allow an individual to explore a terrain for a while and see whether she can find her way around it reliably” (Gardner, 1995b, p. 202). Gardner and his research team spent an entire decade, after the publication of *Frames of Mind*, exploring the plausibility of a MI theory-based “child-centered” learning program. Their most ambitious initiative was the *Spectrum Project*, aimed at creating a museum-like, rich environment for children to deploy their biopsychological potentials (intelligences). A set of 15 learning activities covering seven knowledge domains was created to provide a contextually valid assessment battery of MI intelligence profiles. For instance, to assess *interpersonal intelligence*, children manipulated figures in a scaled-down, 3D replica of their classroom (Chen and Gardner, 2012). The distribution of strengths and weaknesses across the range of intelligences was called the *Spectrum profile*. The ultimate goal was to develop individualized educational interventions adapted to MI intelligence profiles.

However, MI theory does not only posit the existence of eight neurologically plausible intelligences, it also posits that each individual actually *combines* several intelligences to tackle *any* given task, making it unlikely for a test to capture purely specific intelligence strengths and weaknesses (e.g., a test that would isolate bodily-kinesthetic from musical, spatial, and interpersonal intelligences, while observing an individual dancing the tango). Although the 15 assessment tasks from the *Spectrum* battery have been “shown to demonstrate reliability” (Davis et al., 2011, p. 496), valid measures of single or multiple deployment of the eight intelligences are still unsettled:

Direct experimental tests of the [MI] theory are difficult to implement and so the status of the theory within academic psychology remains indeterminate. The biological basis of the theory—its neural and genetic correlates—should be clarified in the coming years. But in the absence of consensually agreed upon measures of the intelligences, either individually or in conjunction with one another, the psychological validity of the theory will continue to be elusive (Davis et al., 2011, p. 498).

Reflecting back on assessment tools for the multiple intelligences, Gardner (2016) admitted that he has “not devoted significant effort to creating such tests” (p. 169). In light of the enormous investment of time and money, he did not want himself to be “in the assessment business” (Gardner, 2011a, p. xiii). Above all, measuring multiple intelligences is inconsistent with Gardner's critique of the traditional IQ theories of intelligence and, for that reason, he shows “reluctance to create a new kind a strait jacket *(Johnny is musically smart but spatially dumb*)” (Gardner, 2011b, p. 5).

Accordingly, the opening survey statement is considered as a neuromyth because of a lack of compelling evidence—mainly due to unsatisfactory measures of MI intelligence profiles—that matching modes of instruction to MI intelligence profiles promotes learning. This intuitively appealing hypothesis, contemplated by Gardner's research team at some point (the *Spectrum Project*) but still open to scientific inquiry, has somehow been taken for granted by laypersons and, over time, embedded into popular culture. In other words, it became a neuromyth.

## Entertaining the “Problematic” Neuromyth

Gardner (2020) blames survey designers for putting up statements “conflating science and practice” and for creating rather than exposing neuromyths. He warns that by “waving the provocative neuromyth flag” with the opening survey statement, the baby (MI theory) might be thrown out with the bathwater (unsubstantiated educational claims derived from it).

First, neither Blanchette Sarrasin et al. (2019) nor other researchers in the field deliberately put up, in their respective surveys, neuromyth statements. Neuromyths are creatures of their own, to be chased, not created. Twenty-five years ago, Gardner (1995b) debunked seven common myths that have grown up from MI theory. Myth #3 (“Multiple intelligences are learning styles”) was so persistent that Gardner (2013) found it necessary to debunk it once again in the new millennium. Survey designers simply exposed yet another, very prevalent myth: Tailoring instruction to pupils' MI intelligence profiles promotes learning.

Second, *any* scientific theory is a potential source of neuromyths. As noted by Geake (2008), the most pervasive neuromyths are ingrained into valid science. Is Roger Sperry's Nobel Prize at stake just because abusive extrapolations of his findings on functional hemispheric lateralization have given rise to one of the most pervasive neuromyths (“left-brained”—“right-brained” people)? By exposing such a popular neuromyth, might the baby (Sperry's contributions to neuroscience) be thrown out with the bathwater? The scientific integrity of MI theory cannot be harmed by the “problematic” neuromyth. Legitimate scientific theories and discoveries are challenged by empirical scrutiny, *not* by false beliefs loosely inspired from them.

Gardner (2020) argues that the way claims are conveyed in neuromyth survey statements (in an all-or-none, true/false fashion) is deceptive. To foster a more constructive dialog between scientists and educators, he advocates that research findings with potential educational implications should be properly *qualified*, in terms of both robustness and caveats. Surprisingly, rather than qualifying the message (the false claim in the opening survey statement), Gardner (2020) shoots the messengers (survey designers). A “more constructive” approach would be (1) to underline the scientific robustness of MI theory—its neurological plausibility (Posner, 2004) *and* (2) to disclose caveats pertaining to direct application of MI theory in educational settings, most notably that research aimed at testing the MI–instruction “matching” hypothesis is still hampered by a lack of consensually agreed upon measures of MI intelligence profiles (Davis et al., 2011). By shooting the messengers rather than qualifying the message (debunking yet another common myth that has grown up from MI theory), Gardner (2020) refrains from pulling the bathtub plug and entertains unsubstantiated educational implications of a legitimate scientific theory of intelligence.

## Author Contributions

The author confirms being the sole contributor of this work and has approved it for publication.

## Conflict of Interest

The author declares that the research was conducted in the absence of any commercial or financial relationships that could be construed as a potential conflict of interest.

## Publisher's Note

All claims expressed in this article are solely those of the authors and do not necessarily represent those of their affiliated organizations, or those of the publisher, the editors and the reviewers. Any product that may be evaluated in this article, or claim that may be made by its manufacturer, is not guaranteed or endorsed by the publisher.
